# Attitudes and perspectives of healthcare workers on treating chronic hepatitis C infection in children and adolescents

**DOI:** 10.3389/fpubh.2024.1504678

**Published:** 2025-01-23

**Authors:** Farihah Malik, Philippa Easterbrook, Giuseppe Indolfi, Claire Thorne

**Affiliations:** ^1^UCL Great Ormond Street Institute of Child Health, University College London, London, United Kingdom; ^2^Department of Global HIV, Hepatitis and STI Programmes, World Health Organization, Geneva, Switzerland; ^3^Meyer Children’s Hospital IRCCS, Florence, Italy; ^4^Department Neurofarba, University of Florence, Florence, Italy

**Keywords:** hepatitis C, child, adolescent, healthcare worker, values, preferences, treatment, DAAs

## Abstract

**Background and aims:**

There are gaps in knowledge about the values and preferences of healthcare workers (HCW) with respect to treatment of children and adolescents living with chronic hepatitis C (HCV) infection. This study was carried out to identify these values and preferences as part of the evidence required to update World Health Organization (WHO) hepatitis C guidelines.

**Methods:**

An online survey was designed and conducted during August/September 2021. Survey questions were developed to address two key questions about treatment of children and adolescents: who to treat, and which direct acting antiviral (DAA) regimens to use. The survey was circulated by the WHO to nine networks providing care to children and adolescents living with HCV infection, with respondents requested to cascade further within their networks.

**Results:**

A total of 137 individuals from 38 countries responded to the survey. There was a trend toward higher preference for treating children of older age groups; 60% of respondents reported a strong preference for treating (i.e., stating they were very likely or likely to treat) children aged 3 to <6 years, 81 and 95% indicated strong preferences for treating those aged 6 to <12 years and 12 to <18 years, respectively. The most preferred DAA regimens for treatment across all age groups were: sofosbuvir/velpatasvir (SOF/VEL), sofosbuvir/ledipasvir (SOF/LDV), and glecaprevir/pibrentasvir (GLE/PIB). These were also reported to be the most commonly available drug regimens at respondents’ facilities.

**Conclusion:**

This survey provides insights from a heterogenous sample of HCWs from across the world with respect to their expressed priorities and preferences for the treatment of children and adolescents with chronic HCV.

## 1 Introduction

In 2019, there were an estimated 58 million persons with chronic HCV infection globally (1), and based on a systematic review conducted in 2019, an estimated 3.26 million (95% uncertainty interval 2.07–3.90) viraemic children and adolescents aged 0–18 years living with chronic HCV infection (2). Short-course, oral, curative direct-acting antiviral (DAA) regimens have transformed treatment for HCV infection in adults. In 2018, WHO recommended a “treat all” approach using one of three recommended pan-genotypic regimens - sofosbuvir/daclatasvir (SOF/DCV), sofosbuvir/velpatasvir (SOF/VEL), or glecaprevir/pibrentasvir (GLE/PIB). For children and adolescents, in the absence of regulatory approval for these DAAs at the time, use of two non-pangenotypic regimens (either sofosbuvir/ledipasvir (SOF/LDV) or sofosbuvir+ribavirin) that had received regulatory approval from the US Food and Drug Administration (FDA) and the European Medicines Agency (EMA) were recommended for use in adolescents (≥12 years) with deferral of treatment for those under 12 years of age pending further data on the use of these regimens for these younger age groups (3).

The predominant mode of acquisition of HCV infection in children is vertical transmission. Older children and adolescents may become infected via unsafe injections and poor infection prevention and control, especially in lower and middle income countries (LMICs) (2, 4). Regardless of age, most children with HCV infection have asymptomatic or minimally symptomatic liver disease, and cirrhosis, hepatocellular carcinoma or extrahepatic manifestations are rare. Yet, recent evidence suggests that those with vertical acquisition developed cirrhosis at an earlier age (5). HCV infection may also decrease the general health and quality of life of adolescents (6, 7). Therefore, early diagnosis and treatment in children is key to preventing long-term morbidity and optimising health (8).

However, until recently, there had been less attention on addressing treatment of HCV in children and adolescents, and no DAA regimens were approved for use in children (9, 10). Since 2018, the high rate of HCV viral clearance observed in adults with the key pangenotypic DAA regimens has been confirmed amongst adolescents and children, leading to regulatory approvals by the FDA and EMA. Amongst adolescents, GLE/PIB, SOF/VEL and sofosbuvir/velpatasvir/voxilaprevir (SOF/VEL/VOX), were approved in 2020 and 2021. Amongst children aged 3 years and older, SOF/LDV was approved in 2019, followed by GLE/PIB and SOF/VEL in 2021.

In 2022 WHO updated their HCV guidelines incorporating expansion of treat all to include all adolescents and children aged 3 years and older. This was based on a systematic review of DAA treatment of children and adolescents with chronic hepatitis C infection (11). To support informed decision-making on the updating of recommendations for children and adolescents, WHO commissioned a values and preferences survey of healthcare workers (HCW) caring for children and adolescents living with HCV infection to ascertain their views and the acceptability of treating children and adolescents and of use of different DAA regimens.

Values and preferences related to the outcomes of an intervention are one of the four main factors that determine the direction and strength of guideline recommendations (12). The other three factors are confidence in the estimates of the effect of the evaluated evidence (i.e., quality of the evidence), the balance of benefits and harms, and resource implications. To formulate a recommendation, the guideline development group considers each factor in turn and judges its importance and effect on the recommendation.

The key objectives of this study were to understand the current practises regarding treatment of children and adolescents in different regions, HCW perspectives on future treatment priorities regarding age at which to treat children and which DAA regimens to use, and to highlight implementation challenges in providing treatment for children and adolescents.

## 2 Methods

### 2.1 Target population and questionnaire development

In consultation with the WHO Paediatric Working Group on Viral Hepatitis, survey questions were developed to establish the values, preferences and acceptability to HCW of three key issues relating to potential new treatment recommendations: which children to prioritise for treatment, which DAA regimens to use, and key service delivery barriers likely to be faced by treatment providers. The survey comprised 17 questions across four sections: (1) information about the respondents, their expertise (specialist or generalist), and practise setting (tertiary, secondary or primary care level), including approximate total number of children living with HCV they had cared for in the past; (2) information about current HCV treatment availability for children and adolescents in their facility and country; (3) priority considerations in decisions to offer treatment to children; eligibility preferences for future HCV treatment and choice of DAA regimens; and (4) programmatic, service delivery and other barriers faced by health care workers in providing access to HCV treatment for children and adolescents and proposed solutions (see Supplementary materials). There were 11 close-ended questions (checkboxes, radio button, Likert scales and grid questions) and six open-ended questions. Responses were anonymised and consent was implied. The online survey was available only in English. However, participants could translate the survey weblink using the automated translation plugins built into web browsers. Survey data were collected and managed using REDCap® electronic data capture tools (13, 14). This study was granted ethics approval by UCL Research Ethics Committee (3,715/006).

### 2.2 Survey dissemination

The survey target group was HCWs, primarily doctors with an interest in and/or experience in caring for children and adolescents living with chronic HCV infection. The survey was circulated through WHO’s six regional viral hepatitis focal persons in the Americas (PAHO), Africa (AFRO), Eastern Mediterranean (EMRO), Europe (EURO), South-East Asia (SEARO) and Western Pacific (WPRO) regional offices for distribution to their regional networks of paediatricians and hepatologists and also through a comprehensive set of global and/or regional networks of paediatric infectious disease specialists or hepatologists likely to be caring for children and adolescents living with HCV infection. The survey opened on 24th August 2021 and closed on 17th September 2021. These networks were:

- Penta child health network^1^
  ○ The PENTAHep consortium – an existing clinical network, established in 2015 including clinicians providing care to children with hepatitis C from organisations across Europe, Africa, and the USA.
  ○ The Penta ID network which spans over 100 clinical sites in 31 countries, including clinical sites and collaborators in Asia, Africa, and the Americas.
- FISPGHAN – Federation of International Societies for Paediatric Gastroenterology, Hepatology and Nutrition and their member organisations at regional levels:
  ○ APPSPGHAN - Asian Pan-Pacific Society for Paediatric, Gastroenterology, Hepatology and Nutrition.
  ○ CAPGAN - Commonwealth Association of Paediatric Gastroenterology, Hepatology and Nutrition.
  ○ ESPGHAN - European Society for Paediatric Gastroenterology, Hepatology and Nutrition.
  ○ LASPHAN - Latin America Society for Paediatric Gastroenterology, Hepatology and Nutrition.
  ○ NASPGHAN - North American Society for Paediatric Gastroenterology, Hepatology and Nutrition.
  ○ PASPGHAN - Pan Arab Society for Paediatric Gastroenterology, Hepatology and Nutrition.

### 2.3 Data cleaning, analysis and definitions

To assess the quality of survey responses, a two-step data cleaning protocol was developed. In the first step, data quality was assessed by *post hoc* automated authentication checks to identify possible duplication of respondents, missing data, straightlining (15), indicators of quick click-throughs, implausible responses and internal consistency checks. Responses were given a flag for each of these data quality issues identified. In the second step, these flagged responses were reviewed manually. An overall data quality score was calculated for each response by adding the number of flags per response, with higher score indicating more data quality concerns. Based on the manual review, responses were excluded from analysis if the data quality score was five or more out of nine possible.

Data summaries and charts were created in Stata (16.1, StataCorp LLC, College Station, TX) and Microsoft Excel (Office 365, version 2,112). Proportions were compared using Fisher’s exact test for categorical variables. Country data were tabulated according to WHO region and World Bank income country classification (16). Text responses to open-ended questions were coded and analysed using a thematic analysis approach by two independent coders (FM and CT). Denominators varied in analyses, depending on the number of respondents answering the question.

## 3 Results

### 3.1 Respondent characteristics

There were 146 survey respondents from 38 different countries, and 137 responses were available for analysis after exclusion of nine incomplete or duplicate entries. Table 1 summarises the characteristics of the survey respondents. Most respondents were from high- (51, 37%) or upper middle-income (49, 36%) countries and according to WHO region from WPRO (58, 42%), PAHO (27, 20%) and EURO (23, 17%).

**Table 1:** Respondent characteristics.

Country income status World Bank income classification	*n* = 137
High income	51 (37%)
Upper middle income	49 (36%)
Lower middle income	27 (20%)
Low income	10 (7%)
WHO region	*n* = 137
AFRO	11 (8%)
EMRO	5 (4%)
EURO	23 (17%)
PAHO	27 (20%)
SEARO	13 (9%)
WPRO	58 (42%)
Clinical roles	*n* = 137
Paediatrician/ paediatric ID specialist/ paediatric hepatologist	69 (50%)
Hepatologist/Gastroenterologist	31 (23%)
ID specialist	18 (13%)
General Physician	10 (7%)
Other/Non-doctor	9 (7%)
Facility level	*n* = 137
Tertiary	110 (80%)
Secondary	14 (10%)
Primary	9 (7%)
Private clinic	3 (2%)
NGO clinic	1 (1%)
Clinical experience	*n* = 136
Less than 1 year	9 (7%)
1–2 years	4 (3%)
3–5 years	14 (10%)
5–10 years	19 (14%)
More than 10 years	90 (66%)

AFRO, WHO African region; EMRO, WHO Eastern Mediterranean region; EURO, WHO European region; ID, Infectious Diseases; NGO, Non-governmental Organisation; PAHO, WHO region of the Americas; SEARO, WHO South East Asia region; WHO, World Health Organization; WPRO, WHO Western Pacific region.

Half of the respondents (69, 50%) identified as either a paediatrician, or a paediatric specialist (paediatric infectious disease (ID) or hepatology), 31 (23%) as either hepatologists or gastroenterologists, and 18 (13%) infectious diseases specialists (Table 1). The majority (110, 80%) of all respondents worked in tertiary centers and 109 (80%) had more than 5 years’ clinical experience.

### 3.2 Treatment experience

Overall, 92 (67%) respondents reported providing care for children or adolescents with HCV infection over the preceding 3 years. The majority had cared for fewer than 25, with a median of five for both adolescents and children, which was highest in the EMRO region at 50 and 100, respectively, and lowest in the AFRO region (0 and 6) (Supplementary Tables S1 and S2). The high number in EMRO region was driven by large caseloads amongst 3 respondents from Egypt with two reporting more than 700 children or adolescents with HCV in their care over the preceding 3 years. A further question examined the regimen used in the 267 treatments used by 80 HCW respondents (Supplementary Table S3). SOF/LDV was the most common treatment regimen used in around one third across all age groups, followed by SOF/VEL in 18%. IFN or PEG-IFN was used in around 29 and 18% of younger and older children, reflecting the only recent regulatory approvals of DAA regimens and lack of access in country.

### 3.3 Treatment availability and funding

Fifty (39%) of 129 respondents reported that no treatment regimens were available at their facility, 50 (39%) had DAAs only, 23 (18%) had access to DAAs and IFN (Supplementary Table S4). Of the 70 sites with DAAs available, the majority were in the EURO, PAHO or WPRO regions. The most common DAA regimens were SOF/LED (*n* = 53), followed by SOF/VEL (*n* = 36), GLE/PIB (*n* = 33) and SOF/DCV (*n* = 17) (Supplementary Table S5).

The main sources of funding for paediatric hepatitis treatment at respondents’ facilities were through the government or public sector (53%, 73/137) and self-funded (i.e., by families) (31%, 42/137); a smaller proportion stated private insurance 7% (*n* = 10), NGO 1% (*n* = 1) and other 6% (*n* = 8) funding as the main funding source.

### 3.4 Outcomes considered by HCWs when deciding whether to treat children or adolescents

**Figure 1:**
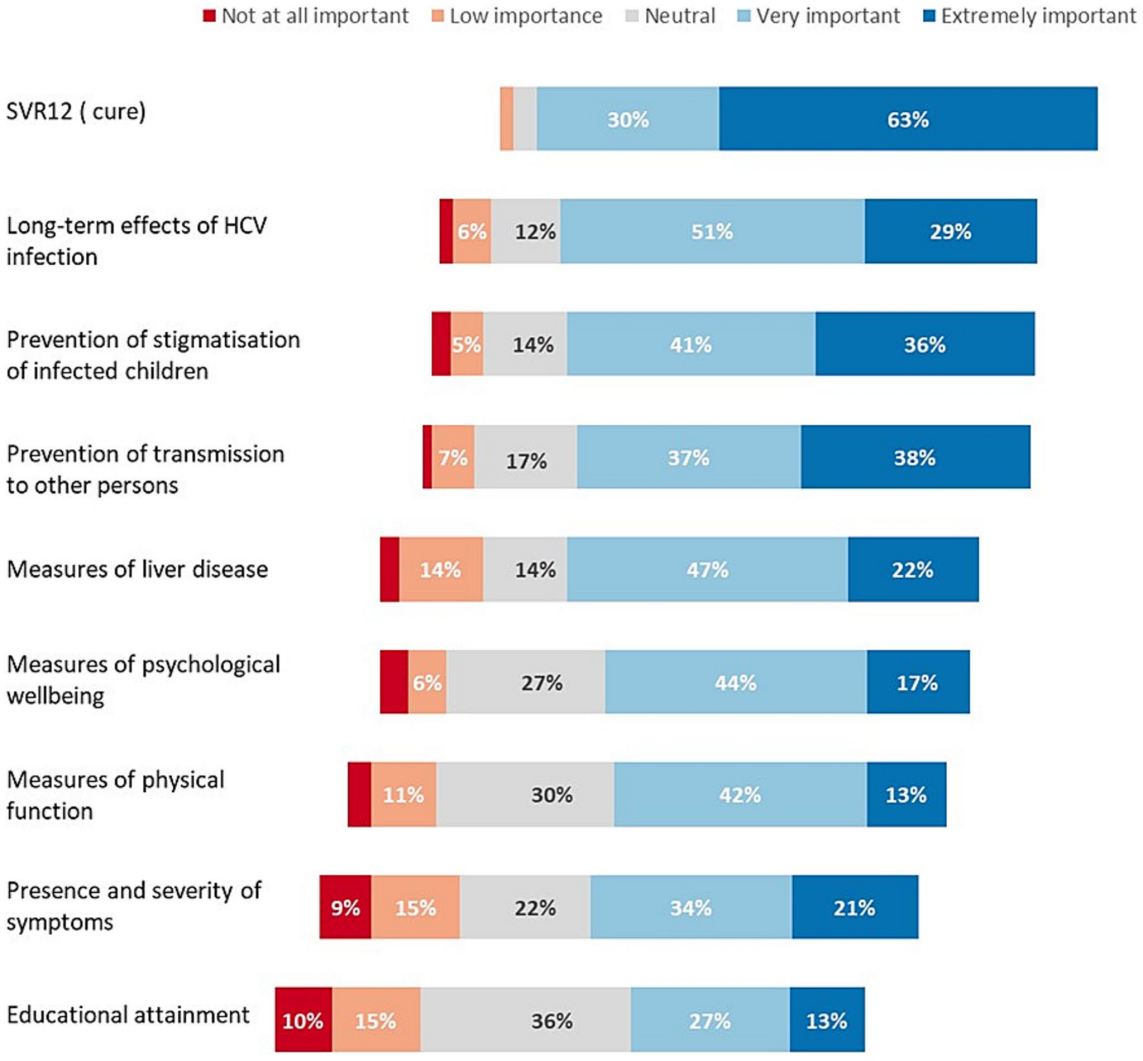
Respondents’ key considerations when deciding whether to treat children and adolescents.

Respondents rated nine considerations in deciding to treat children and adolescents (Figure 1). The following were rated as extremely important by more than one third of respondents: attainment of HCV cure (i.e., SVR12) (63%), reducing long-term adverse effects of HCV infection (29%), prevention of stigmatisation of children with chronic infection (36%), and prevention of transmission to others (38%) (Figure 1). Other outcomes that were ranked lower were: extent of liver disease, measures of psychological wellbeing or physical function, severity of symptoms and impact on educational attainment.

### 3.5 HCW preferences on age to treat

In response to the question “*In your opinion, what is the threshold age at which treatment of children with HCV should be recommended in treatment guidelines?*,” 60% of respondents (81/137) supported the initiation of treatment at either ≥3 years or for all age groups in the future guideline recommendations, 20 (15%) at ≥6 years, and 29 (21%) only for adolescents ≥12 years.

**Figure 2:**
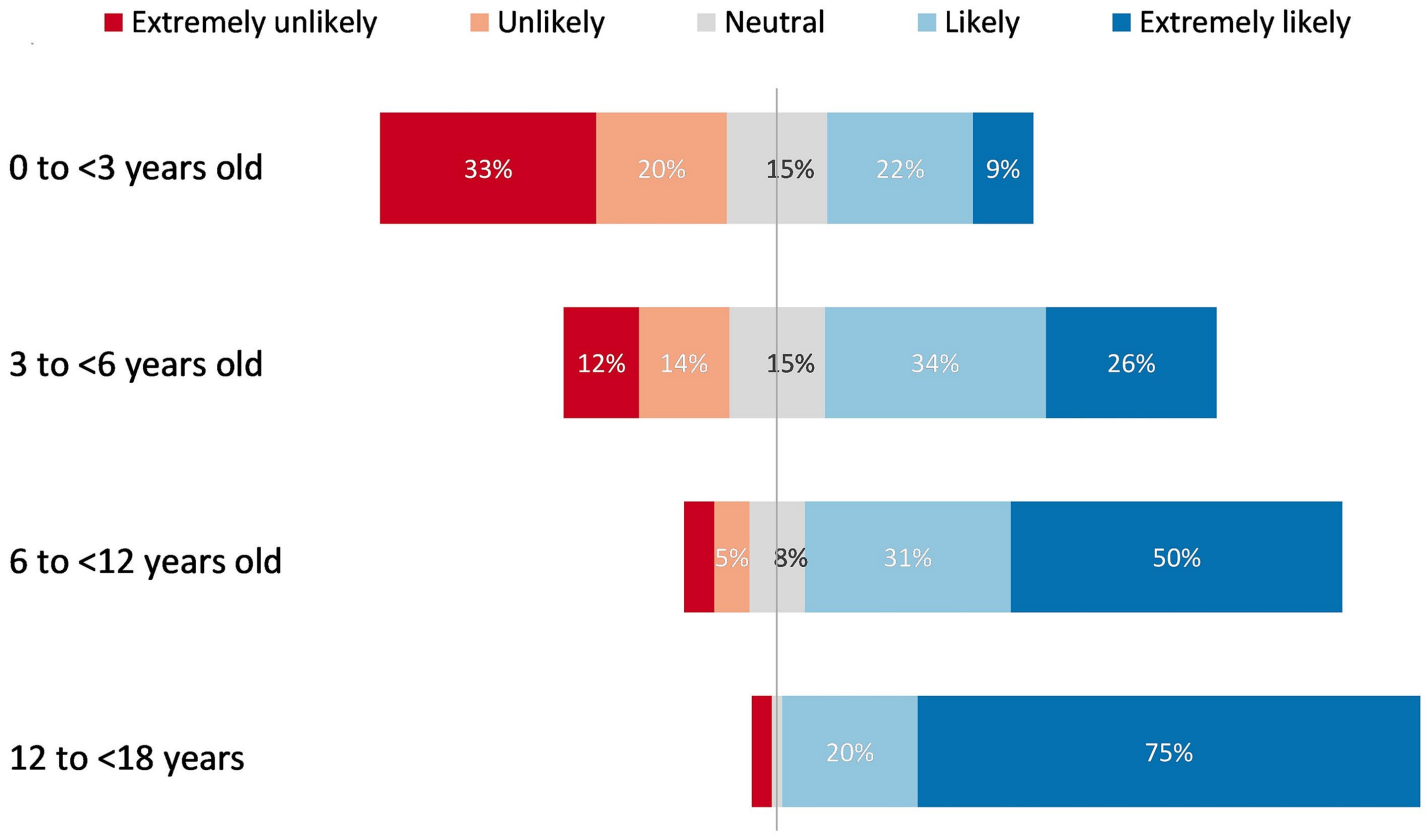
Respondents’ preferences to treat children in different age groups if DAAs were available for paediatric HCV treatment.

In response to the Likert scale question “*If DAAs were available for treatment of HCV infection in children and adolescents in your practice/facility, how likely would you be to treat children in the following age groups*,” respondents showed a clear trend toward greater preference for treating older age groups, with the majority of the respondents stating they would be likely to treat children above 6 years of age (Figure 2). Ninety-five percent (127/133) reported a strong preference (75% extremely likely and 20% likely) to treat adolescents aged 12 to <18 years; 81% (107/131) reported a strong preference (50% extremely likely and 31% likely) to treat older children aged 6 to <12 years; 60% (78/130) reported a strong preference (26% extremely likely and 34% likely) to treat younger children aged 3 to <6 years; and 31% (41/130) reported a strong preference (9% extremely likely and 22% likely) to treat children aged <3 years.

The main reasons given for being less likely to treat younger aged children included the more benign nature of disease in childhood, side effects of treatment and difficulties with drug administration, and lack of DAA approvals.

The most commonly reported reason for not treating the youngest group of children, i.e., those below 3 years old was due to the possibility of clearance in this age group. There were differences in respondents’ opinions regarding the age up to which spontaneous clearance can occur, with some advocating waiting until the child is older than 6 or even 12 years old to initiate treatment.

Several participants reported that given the asymptomatic nature of disease in early childhood and relatively mild liver disease experienced by young children, treatment of HCV in childhood was not a priority nor a matter of urgency. Participants’ responses indicated the underlying belief that the primary goal of therapy is to treat liver disease or fibrosis rather than eliminating HCV, improving HCV-related health outcomes, or reducing transmission of HCV to others. Since children often do not develop fibrosis in childhood, respondents were of the opinion that treatment can wait till they are older.

Many respondents expressed concerns regarding potential and perceived side effects of DAA treatment for young children, including those who had suggested that treatment be recommended for children of all age groups. Some respondents also reported a lack of information on long-term side effects particularly regarding the impact of DAA treatment on puberty and growth.

With regards to treatment of young children, respondents expressed concerns about difficulties with administering DAA tablets, given the unavailability of suitable paediatric formulations, suggesting that they would prefer to defer treatment until children are old enough to be able to reliably swallow tablets. Some respondents indicated that young children’s inability to swallow DAAs might pose challenges for treatment adherence.

Several respondents reported that their preference to treat was based on national guidelines and policies governing paediatric DAA use as well as availability of DAAs within their countries. Respondents from LMICs also raised concerns about the affordability of DAAs, particularly low-cost formulations suitable for paediatric use. In anticipation of the unavailability of low-cost paediatric formulations, one respondent suggested HCV treatment during pregnancy to prevent vertical transmission to the newborn child.

To support their decision to not treat the youngest group of children (less than 3 years old), some respondents reported lack of, or more limited, clinical trial data on DAA safety and efficacy in this age group. Some cited the paucity of paediatric clinical trials in their countries, alluding to the need for local trial data as a requirement for approving or registering medications within their countries.

The third emerging theme related to the HCWs’ prior treatment experience and their readiness to provide DAA treatment now, if approved and available. Some respondents indicated hesitancy to treat children because they lacked experience or, were not authorised to treat paediatric HCV.

### 3.6 Other factors affecting HCWs’ preferences for prioritising paediatric patients to treat

Respondents were asked which children they would prioritise for treatment with DAAs and who they would not wish to treat with DAAs. The most common theme that emerged from responses to these two questions was age-based prioritisation, with respondents indicating that they would prioritise treatment for older children but would prefer not to treat younger ones. One paediatrician reiterated their concern about effect of DAA treatments on growth in pre-pubertal children and reported they would wait for more long-term follow-up data on growth outcomes before treating this age group.

Another common theme across both these questions was a treat all approach with respondents stating that they “would treat all infected children” and “would not prioritise - I would treat as soon as is feasible.”

Many respondents reported that whilst they would recommend treatment “for all children for whom DAAs are licenced,” and that “all infected children should be treated,” they would prioritise treatment for “children with symptoms,” those with evidence of fibrosis or severe liver disease, HCV/HIV co-infection, haematological diseases, cancer, immunosuppression, renal disease, extra-hepatic manifestations, evidence of fibrosis or severe liver disease.

With regards to the groups of children that HCWs would not wish to treat, some respondents (*n* = 6, 7%) indicated not wishing to treat children “with fatal comorbid conditions” where the comorbidities are “life limiting,” whilst some others (*n* = 7, 8%) specified that they would not wish to treat children at “high risk for poor treatment adherence,” or “who cannot take medication PO [orally], or are unable to adhere to DAAs, e.g., due to “non-compliant parents.”

### 3.7 HCWs’ preferences for specific DAA regimens

In response to the question *“If DAAs were available for treatment of HCV infection in children and adolescents in your practice/facility, which DAA regimens would you prefer to use in the following age groups*,” the majority of 113 respondents (74%, 84/113) preferred to use DAA only regimens for treatment across all paediatric age groups, but 19% (21/113) indicated the option for use of interferon or DAAs, depending on availability.

The most preferred DAA regimens for treatment across all age groups were SOF/VEL (61%), SOF/LDV (56%) and GLE/PIB (50%), followed by SOF/DCV (28%) and SOF/VEL/VOX (24%) (Table 2).

**Table 2:** Respondents’ (*n* = 113) preferences for HCV treatment, by drug regimen and age group (number and percentage).

	0 to < 3 years	3 to < 6 years	6 to < 12 years	12 to < 18 years	Total*
SOF + IFN	4	8	10	12	18 (16%)
SOF + RBV	4	12	17	18	26 (23%)
SOF/DCV	3	8	13	31	32 (28%)
SOF/LDV	7	31	42	60	63 (56%)
SOF/VEL	11	27	43	67	69 (61%)
SOF/VEL/VOX	2	7	14	27	27 (24%)
GLE/PIB	9	25	29	55	57 (50%)
Total*	26 (23%)	66 (58%)	81 (72%)	113 (100%)	

*Percentages do not add up to 100 as respondents could select multiple options.

DCV, Daclatasvir; GLE, Glecaprevir; IFN, Interferon; LDV, Ledipasvir; PIB, Pibrentasvir; RBV, Ribavirin; SOF, Sofosbuvir; VEL, Velpatasvir; VOX, Voxilaprevir.

Amongst the 83 HCW who provided reasons for preferring a particular drug regimen, the most common were treatment availability (*n* = 21, 25%), DAA safety (17, 20%), included in national guideline recommendations (17, 20%), treatment efficacy (12, 17%), pangenotypic regimen (11, 13%), short treatment durations (10, 12%) and affordability (7, 8%).

### 3.8 Addressing programmatic gaps and service delivery barriers

**Figure 3:**
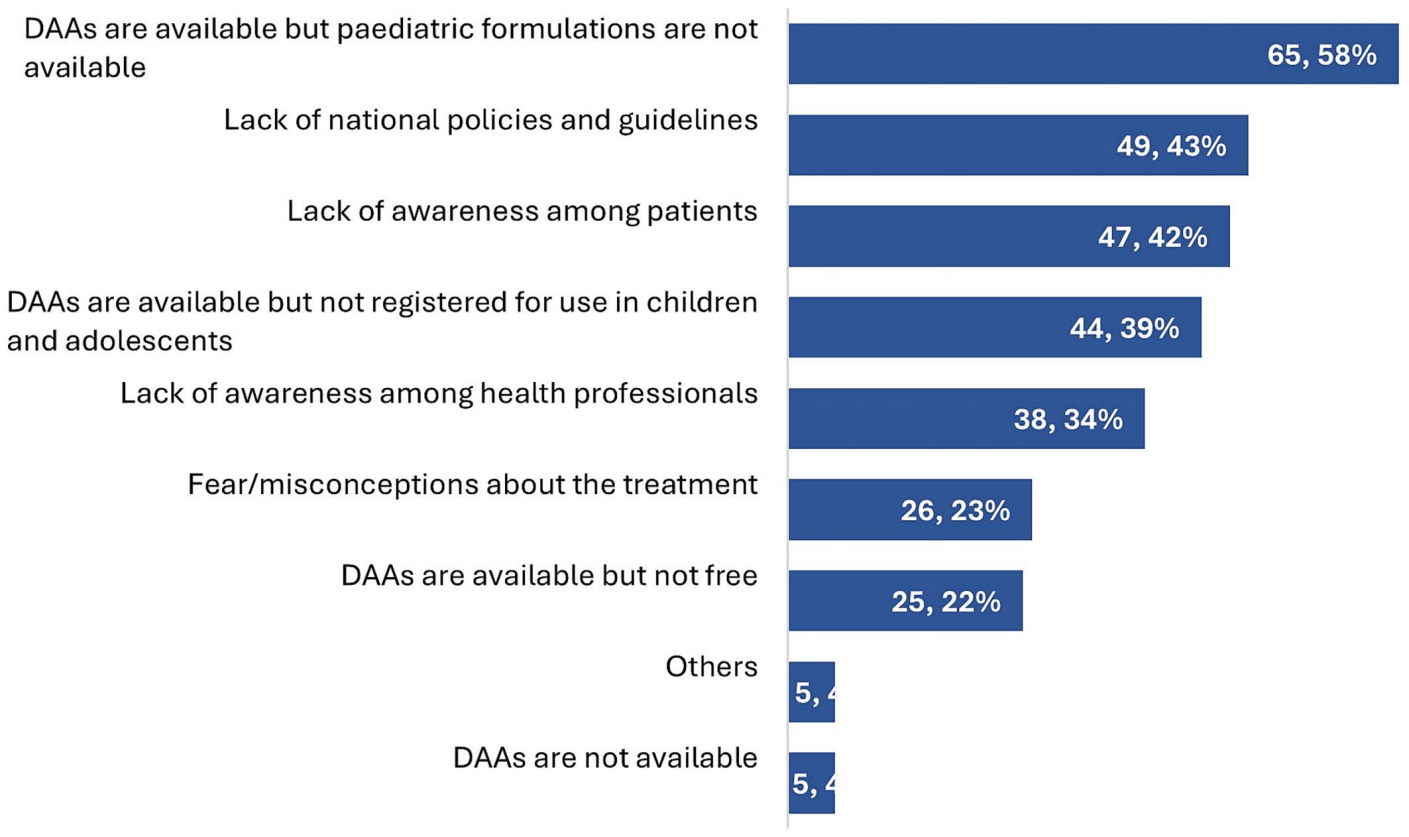
Barriers faced by HCWs in treating children and adolescents with HCV infection (*n* = 113).

The main service delivery barriers in paediatric HCV treatment identified by the respondents were the unavailability of paediatric DAA formulations (65/113, 58%), lack of national policies and guidelines recommending treatment (49/113, 43%), lack of awareness of treatment amongst patients and parents (47/113, 42%), and DAAs not being registered for use in paediatric populations (44/113, 39%) (Figure 3).

Respondents’ suggestions to address these barriers focused on DAAs (including cost, availability, paediatric formulation, and registration for paediatric use), awareness-raising (both amongst patients, parents or caregivers and HCWs), and the need for national health authorities to develop policies and clinical guidelines to include testing and treatment for paediatric HCV infection. Additionally, several respondents also identified the need for improving diagnostic algorithms to include paediatric case-finding approaches and improve testing for children and adolescents.

Respondents suggested that paediatric formulations would improve treatment uptake as “for youngest children, syrup formulas would make the treatment easier.” Even respondents from HICs who had access to DAAs for paediatric treatment highlighted facing “difficulty in access to paediatric formulations” and emphasised the need for “expedited roll out of new formulations for children.” Respondents from LMICs also echoed this need whilst emphasising that paediatric formulations should be affordable and available at low cost.

## 4 Discussion

Values and preferences surveys are an important component of the evidence reviewed in guideline development as they describe the relative importance assigned to health outcomes by stakeholders affected by them, how such importance varies within and across populations, and whether this importance or variability is surrounded by uncertainty (12). The less uncertainty or variability there is about the values and preferences of people experiencing the critical or important outcomes, the greater the likelihood of a strong recommendation. Several such surveys of various stakeholder groups have been conducted to inform WHO’s recommendations across different disease areas (12, 17–19). These stakeholders include patients, caregivers, and the HCW responsible for providing health services and implementing the recommendations. This survey was conducted to assess HCW perspectives on treatment of children and adolescents with HCV infection to inform updated WHO hepatitis C treatment guidelines (1). In 2022, WHO issued new recommendations on expanding treatment to adolescents and children with chronic HCV infection based on systematic review of the evidence including results from this survey, as well as HCV simplified service delivery and diagnostics (1).

Findings of this values and preferences survey indicate strong support for treatment of adolescents and children overall, with this highest for the oldest age groups: 95% respondents stated that they would be likely to treat adolescents aged ≥12 years, 81% to treat children above 6 years of age and 60% those from aged 3 to 6 years. Overall, 60% supported recommending treatment either for children of all ages or for those >3 years old. These results are in line with the previously reported results from a survey of European paediatricians, where respondents also indicated an age-based preference with a stronger inclination to treat older children (20). In the updated recommendations, WHO recommends HCV treatment for all adolescents and children aged 3 years or greater (1).

This age-based preference to treat may in part reflect HCWs’ experience, as most HCWs had treated adolescents (81%) whilst fewer reported having treated younger children. Although DAAs have been approved by regulators for the treatment of young children above 3 years old since August 2019, almost 2 years before this survey was conducted, only a third of respondents with any paediatric treatment experience had treated patients in the 3 to <6 years old age group.

Respondents’ reasons for not treating younger children included absence of symptoms and minimal disease progression, a limited evidence-base and significant rate of spontaneous clearance in young children. However, there were differing opinions about the age range when spontaneous clearance can occur in vertically infected children. Recent individual patient data meta-analyses from the largest purely prospective dataset assembled indicate a marked fall in clearance rates with age, with most clearance occurring before 3 years of age (21).

The more limited evidence-base on DAAs in young children (3 to <6 years) is reflected in the results of a WHO-commissioned systematic review and meta-analysis of the efficacy and safety of key DAA regimens for adolescents (12–18 years), older children (6–11 years) and younger children (3–5 years) with chronic hepatitis C virus infection, based on the same age groupings used in the trials for regulatory approval. Together, they reported treatment experience in 1891 adolescents (35 study arms), 472 older children (13 study arms) and 167 younger children (7 study arms). There were no placebo-controlled randomised controlled trials, and findings were based on summary estimates of sustained virological response cure rates by regimen in the three age groups (11). Of note, there were no data on SOF/DCV use for treatment of young children (11).

Another important consideration that HCWs in the survey likely factored in when reporting their preferences for who to treat and who to prioritise was the ease of administering the currently available formulations. The unavailability of formulations suitable for paediatric use was identified by 58% of the respondents as a barrier to service delivery, whilst the systematic review demonstrated that five of the 10 treatment discontinuations were in young children (3 to <6-year-olds) and related to issues of drug palatability (11). Real-world implementation of tablet formulations to young children could result in more palatability-related treatment discontinuations especially in settings without adequate clinical support.

This survey also provides an assessment as of 2021 on DAA availability and utilisation. Findings indicate that paediatric HCV treatment is limited to specialist providers based at tertiary care centres. SOF/LDV was reported to be the most commonly available regimen across facilities and most used by HCWs, followed by GLE/PIB and SOF/VEL. SOF/DCV was reported to be used mainly by HCWs from LMICs. For effective roll out of DAAs for children, there is a need to understand these gaps in implementation using more in-depth, country-specific approaches as the lack of availability of paediatric formulations is a recurring problem for several diseases, not just hepatitis C.

HCWs’ preferences for treatment largely reflected access to certain regimens based on regulatory approval and country registration arrangements. Respondents’ preferred pangenotypic DAA regimens (SOF/VEL, SOF/DCV and GLE/PIB) for treatment across all paediatric age groups. – These pangenotypic regimens are recommended in the updated WHO recommendations (1). SOF/LDV was one of the most commonly used DAA regimens in the survey, likely reflecting it being the first regimen approved for paediatric use but is not pangenotypic. Around the time of the survey, there was FDA and EMA approval for GLE/PIB by mid-2021 in children 3 years old and above, and SOF/VEL by November 2021 in children 3 years old and above.

The finding that HCW reported the lack of national policies or guidelines recommending DAA treatment for children and/or lack of registration of DAAs for paediatric treatment in their country as barriers to treatment underscores results from a previous global review of national HCV policies (10), that found that only 27% of WHO member states had policy recommendations for treating children.

The value of this survey lies in the global overview it provides of HCWs’ perspectives on treating adolescents and children with HCV infection and insights into treatment decision-making. It also addresses the DAA availability for paediatric treatment in 2021 and the gaps in implementation of paediatric DAA treatment programs. There are several limitations to this survey. First, it was undertaken in 2021 prior to the registration of key DAAs with major regulatory authorities, and thus is based on HCWs’ experience in some countries (especially in LMICs) at a time prior to availability of these DAAs. Second, the survey network reached individuals with an interest in HCV especially those in tertiary centers, and therefore cannot be considered representative of all HCWs likely to be treating HCV in children. Third, this survey was focused on the perspectives of HCWs and did not include an assessment of end-user (parents and children) perspectives.

With the WHO treatment guidelines now having been updated to include treatment of all adolescents and children aged 3 years and older, the next step is to ensure national guidelines are revised to reflect these new recommendations, and to ensure wide dissemination of these guidelines to HCW and awareness campaigns for parents and carers to inform them of treatment availability. Improving availability and uptake of paediatric HCV treatment requires undertaking forecasting and procurement and further implementation research to identify which service delivery approaches are most effective in different contexts to ensure effective case finding. The results provide policymakers and implementers with information about barriers to paediatric HCV treatment, and the findings emphasise a need for child friendly DAA formulations to be approved and registered at national level and available at low-cost to patients.

## Data Availability

The original contributions presented in the study are included in the article/Supplementary materials, further inquiries can be directed to the corresponding author/s.

## Footnotes

^1^ https://penta-id.org/
