# Supplementary material for: Attitudes and perspectives of healthcare workers on treating chronic hepatitis C infection in children and adolescents
Source: Front Public Health. 2025 Jan 23;12:1504678. doi: 10.3389/fpubh.2024.1504678 (PMC11798806; doi:10.3389/fpubh.2024.1504678)
Supplement: Supplementary file 1 [file Table_1.pdf]

Malik F, Easterbrook P, Indolfi G and Thorne C (2025) Attitudes and perspectives of healthcare workers on treating chronic hepatitis C infection in children and adolescents.

Table S1 - Distribution of paediatric HCV patients in care per respondent, by WHO region

	AFRO (n=6)	EMRO (n=5)	EURO (n=22)	PAHO (n=18)	SEARO (n=5)	WPRO (n=36)	Total (n=92)
**Children (<12 years)**							
Median (Q1, Q3)	0 (0, 5)	50 (10, 300)	9 (5, 20)	5 (2, 25)	3 (1, 5)	3 (1, 5)	5 (2, 10)
Min, Max	0, 10	6, 500	0, 50	0, 124	0, 10	0, 60	0, 500
**Adolescents (12-18 years)**							
Median (Q1, Q3)	6 (5, 10)	100 (50, 250)	13 (7, 18)	6 (5, 15)	10 (2, 10)	3 (1, 5)	5 (3, 15)
Min, Max	2, 20	10, 700	0, 50	1, 55	0, 80	0, 150	0, 700

AFRO: Africa, AMRO: the Americas, EMRO: Eastern Mediterranean, EURO: Europe, SEARO: South-East Asia, WHO: World Health Organization, WPRO: Western Pacific
