# Supplementary material for: Attitudes and perspectives of healthcare workers on treating chronic hepatitis C infection in children and adolescents
Source: Front Public Health. 2025 Jan 23;12:1504678. doi: 10.3389/fpubh.2024.1504678 (PMC11798806; doi:10.3389/fpubh.2024.1504678)
Supplement: Supplementary file 2 [file Table_2.pdf]

Table S2 - Treatment experience of healthcare workers (n=80): number and percentage of healthcare workers, by number of patients treated and age group*

	Number of paediatric patients treated for HCV				
**Age group**	**1 – 10**	**11 – 20**	**21 – 50**	**More than 50**	**Total† (n=80)**
**0 to <3 years old**	1	0	0	0	1 (1%)
**3 to <6 years old**	27	2	0	0	29 (36%)
**6 to <12 years old**	31	6	3	1	41 (51%)
**12 to <18 years**	42	12	6	5	65 (81%)

*Outside clinical trials
Column percentages do not add up to 100 as participants could select multiple options to this question.
† Number of respondents treating paediatric patients in each age group (% of total).

Malik F, Easterbrook P, Indolfi G and Thorne C (2025) Attitudes and perspectives of healthcare workers on treating chronic hepatitis C infection in children and adolescents.
