# Supplementary material for: Attitudes and perspectives of healthcare workers on treating chronic hepatitis C infection in children and adolescents
Source: Front Public Health. 2025 Jan 23;12:1504678. doi: 10.3389/fpubh.2024.1504678 (PMC11798806; doi:10.3389/fpubh.2024.1504678)
Supplement: Supplementary file 3 [file Table_3.pdf]

Table S3 - Drug regimens used for paediatric HCV treatment: number and percentage† of healthcare workers (n=80), by drug regimen and age group*

	0 to <3 years n= 1	3 to <6 years n= 29	6 to <12 years n= 41	12 to <18 years n= 65	Total prescribed treatments n= 267
**IFN or Peg-IFN + RBV**	0	13 (45%)	14 (34%)	9 (14%)	**36 (13%)**
**SOF + IFN**	0	2 (7%)	3 (7%)	6 (9%)	**11 (4%)**
**SOF + RBV**	0	4 (14%)	12 (29%)	13 (20%)	**29 (11%)**
**SOF/DCV**	0	0	3 (7%)	19 (29%)	**22 (8%)**
**SOF/LDV**	1 (100%)	14 (48%)	23 (56%)	42 (65%)	**80 (30%)**
**SOF/VEL**	0	7 (24%)	15 (37%)	26 (40%)	**48 (18%)**
**SOF/VEL/VOX**	0	1 (3%)	2 (5%)	7 (11%)	**10 (4%)**
**GLE/PIB**	0	4 (14%)	8 (20%)	19 (29%)	**31 (12%)**
					**267**

*Outside clinical trials
Column percentages are reported with the number of HCWs reporting having treated a particular age group as the denominator.
† Cell percentages do not add up to 100 as participants could select multiple options to this question and are expressed as proportion of respondents having used the regimen, among those treating children within the specified age group (column totals).

DAA- direct acting antiviral; DCV- Daclatasvir; GLE- Glecaprevir; IFN- Interferon; LDV- Ledipasvir; PEG-IFN- Pegylated interferon; PIB- Pibrentasvir; RBV- Ribavirin; SOF- Sofosbuvir; VEL- Velpatasvir; VOX-Voxilaprevir
