# Supplementary material for: Attitudes and perspectives of healthcare workers on treating chronic hepatitis C infection in children and adolescents
Source: Front Public Health. 2025 Jan 23;12:1504678. doi: 10.3389/fpubh.2024.1504678 (PMC11798806; doi:10.3389/fpubh.2024.1504678)
Supplement: Supplementary file 4 [file Table_4.pdf]

**Table S4 - Characteristics of respondents (n=129) countries and facilities, by type of paediatric HCV treatments available at facilities**

| | No treatments | IFN only | Both IFN and DAAs | DAAs only |
|---|---|---|---|---|
| Number of respondents: | n= 50 | n= 6 | n= 23 | n= 50 |
| **WHO Region** | | | | |
| AFRO | 4 (8%) | 0 | 0 | 6 (12%) |
| EMRO | 0 | 0 | 1 (4%) | 4 (8%) |
| EURO | 2 (4%) | 1 (17%) | 9 (39%) | 10 (20%) |
| PAHO | 9 (18%) | 0 | 5 (22%) | 12 (24%) |
| SEARO | 11 (22%) | 0 | 0 | 2 (4%) |
| WPRO | 24 (48%) | 5 (83%) | 8 (35%) | 16 (32%) |
| **World Bank income classification** | | | | |
| High income | 10 (20%) | 2 (33%) | 15 (65%) | 23 (46%) |
| Upper middle income | 24 (48%) | 4 (67%) | 6 (26%) | 11 (22%) |
| Lower middle income | 11 (22%) | 0 | 2 (9%) | 11 (22%) |
| Low income | 5 (10%) | 0 | 0 | 5 (10%) |
| **Type of facility** | | | | |
| Tertiary | 32 (64%) | 6 (100%) | 21 (91%) | 44 (88%) |
| Secondary | 10 (20%) | 0 | 2 (9%) | 1 (2%) |
| Primary | 6 (12%) | 0 | 0 | 3 (6%) |
| Private clinic | 1 (2%) | 0 | 0 | 2 (4%) |
| NGO clinic | 1 (2%) | 0 | 0 | 0 |

AFRO- WHO African region; DAA- direct acting antiviral; EMRO- WHO Eastern Mediterranean region; EURO- WHO European region; IFN- Interferon; PAHO- WHO region of the Americas; SEARO- WHO South East Asia region; WPRO- WHO Western Pacific region
