# Supplementary material for: Attitudes and perspectives of healthcare workers on treating chronic hepatitis C infection in children and adolescents
Source: Front Public Health. 2025 Jan 23;12:1504678. doi: 10.3389/fpubh.2024.1504678 (PMC11798806; doi:10.3389/fpubh.2024.1504678)
Supplement: Supplementary file 5 [file Table_5.pdf]

Table S5 - Characteristics of respondents' (n=70) countries and facilities, by type of DAA-only regimens available at facilities

Seventy respondents provided details of the DAA-only treatment regimens available at their facilities.

	SOF/DCV (n= 17)	SOF/LDV (n= 53)	SOF/VEL (n= 36)	SOF/VEL/VOX (n= 6)	GLE/PIB (n= 33)
**WHO Region**					
AFRO	5 (29%)	2 (4%)	4 (11%)	1 (17%)	0
EMRO	2 (12%)	3 (6%)	1 (3%)	0	0
EURO	3 (18%)	17 (32%)	7 (19%)	0	15 (45%)
PAHO	2 (12%)	14 (26%)	11 (31%)	3 (50%)	9 (27%)
SEARO	2 (12%)	0	2 (6%)	0	0
WPRO	3 (18%)	17 (32%)	11 (31%)	2 (33%)	9 (27%)
**World Bank income classification**					
High income	4 (24%)	33 (62%)	19 (53%)	3 (50%)	31 (94%)
Upper middle income	4 (24%)	11 (21%)	9 (25%)	2 (33%)	1 (3%)
Lower middle income	5 (29%)	8 (15%)	5 (14%)	0	1 (3%)
Low income	4 (24%)	1 (2%)	3 (8%)	1 (17%)	0
**Type of facility**					
Tertiary	13 (76%)	50 (94%)	33 (92%)	6 (100%)	31 (94%)
Secondary	1 (6%)	2 (4%)	1 (3%)	0	1 (3%)
Primary	1 (6%)	1 (2%)	2 (6%)	0	1 (3%)
Private clinic	2 (12%)	0	0	0	0
NGO clinic	0	0	0	0	0

Percentages are expressed as a proportion of the column totals.

AFRO- WHO African region; DAA- direct acting antiviral ; DCV- Daclatasvir; EMRO- WHO Eastern Mediterranean region; EURO- WHO European region; GLE- Glecaprevir; LDV- Ledipasvir; PAHO- WHO region of the Americas; PIB- Pibrentasvir; RBV- Ribavirin; SEARO- WHO South East Asia region; SOF- Sofosbuvir; VEL- Velpatasvir; VOX- Voxilaprevir; WPRO- WHO Western Pacific region
