# Supplementary material for: Attitudes and perspectives of healthcare workers on treating chronic hepatitis C infection in children and adolescents
Source: Front Public Health. 2025 Jan 23;12:1504678. doi: 10.3389/fpubh.2024.1504678 (PMC11798806; doi:10.3389/fpubh.2024.1504678)
Supplement: Supplementary file 6 [file Supplementary_file_1.pdf]

**Survey questionnaire**

# Treatment of chronic hepatitis C infection in children and adolescents

## Values and preferences survey

**Survey**

You are being invited to participate in an online survey about treatment for children and adolescents living with chronic hepatitis C virus (HCV) infection. This survey is being conducted to understand healthcare workers' preferences about which children to treat and what direct acting antiviral (DAA) regimens to use. Results from this survey will be used to update World Health Organization's (WHO) guideline recommendations on treatment of children (those < 12 years old) and adolescents (between 12 and 18 years old).

The survey comprises 17 questions and should take no more than 15 minutes to complete.

Survey information is anonymised, and no personal identifiers will be associated with your responses or any final reports of these data. Your participation is entirely voluntary. Further details, including information about data protection, are available in the participant information sheet that you can download here.

For further information, please contact farihah.malik.18@ucl.ac.uk

We very much appreciate your time in completing this important survey.

**Survey Questions**

| **Section A Information about you and your practice** | |
|---|---|
| 1. Please type the country for which you are most qualified to discuss current practices for HCV treatment (in most cases, will be your country of residence) | Open ended |
| 2. What best describes your role?<br><br>Please select one | Dropdown menu with the following options:<br>☐ Hepatologist/ Gastroenterologist<br>☐ Infectious disease specialist<br>☐ Paediatrician/ paediatric infectious diseases/paediatric hepatologist<br>☐ General Physician/ Family physician<br>☐ Nurse<br>☐ Other (please specify) |
| 3. At what type of facility do you practice?<br><br>Please select one | ☐ Tertiary (e.g. national, regional or university hospital)<br>☐ Secondary (e.g. district or county hospital)<br>☐ Primary (e.g. community hospital or rural centre)<br>☐ Private clinic<br>☐ NGO clinic |
| 4. How long have you been working in the field of viral hepatitis? | ☐ Less than 1 year<br>☐ 1-2 years<br>☐ 3-5 years<br>☐ 5-10 years<br>☐ More than 10 years |
| 5. Approximately how many children and adolescents with chronic HCV have you cared for **in your practice or facility over the last three years**?<br><br>Please provide estimates | Children (<12 years)<br><br>Adolescents (12-18 years)<br><br>[SKIP - If both are zero skip to Q6] |

5a. Approximately how many children and adolescents with HCV have you treated **over the last three years** in your practice/facility, in the following different age groups (outside of clinical trials)?

Please check all that apply

| | None | 1 – 10 | 11 – 20 | 21 – 50 | More than 50 |
|---|---|---|---|---|---|
| 0 to < three years old | | | | | |
| 3 to < 6 years old | | | | | |
| 6 to < 12 years old | | | | | |
| 12 to <18 years | | | | | |

5b.**Over the last three years**, what drug regimens have you used to treat children and adolescents with HCV in your practice/facility, in the following different age groups?

Please check all that apply

| | 0 to < three years old | 3 to < 6 years old | 6 to < 12 years old | 12 to <18 years |
|---|---|---|---|---|
| Interferon or Pegylated interferon + ribavirin | | | | |
| Sofosbuvir +interferon | | | | |
| Sofosbuvir + ribavirin | | | | |
| Sofosbuvir/daclatasvir | | | | |
| Sofosbuvir/ledipasvir | | | | |
| Sofosbuvir/velpatasvir | | | | |
| Sofosbuvir/velpatasvir/voxilaprevir | | | | |
| Glecaprevir/pibrentasvir | | | | |

**Section B Information about current HCV treatment for children and adolescent in your practice/facility and country**

6. Are any drug regimens currently available **in your practice/facility** for treatment of HCV infection in children and adolescents?

☐ Yes
☐ No [Skip to Q7]

6a. Which of the following drug regimens are **currently available in your practice/facility *** for treatment of HCV infection in adolescents and children, in the following different age groups?
*outside of clinical trials

| | 0 to < three years old | 3 to < 6 years old | 6 to < 12 years old | 12 to <18 years |
|---|---|---|---|---|
| Interferon or Pegylated interferon + ribavirin | | | | |
| Sofosbuvir + interferon | | | | |
| Sofosbuvir + ribavirin | | | | |

| | | | | |
|---|---|---|---|---|
| Sofosbuvir/daclatasvir | | | | |
| Sofosbuvir/ledipasvir | | | | |
| Sofosbuvir/velpatasvir | | | | |
| Sofosbuvir/velpatasvir/voxilaprevir | | | | |
| Glecaprevir/pibrentasvir | | | | |

7Are any drug regimens currently registered and approved **in your country** for treatment of HCV infection in children and adolescents?

☐ Yes
☐ No [Skip to Q8]
☐ Don't know [Skip to Q8]

7a.Which of the following drug regimens are currently **registered and approved** for treatment of HCV infection in children and adolescents **in your country**, in the following different age groups?

| | 0 to < three years old | 3 to < 6 years old | 6 to < 12 years old | 12 to <18 years |
|---|---|---|---|---|
| Interferon or Pegylated interferon + ribavirin | | | | |
| Sofosbuvir + interferon | | | | |
| Sofosbuvir + ribavirin | | | | |
| Sofosbuvir/daclatasvir | | | | |
| Sofosbuvir/ledipasvir | | | | |
| Sofosbuvir/velpatasvir | | | | |
| Sofosbuvir/velpatasvir/voxilaprevir | | | | |
| Glecaprevir/pibrentasvir | | | | |

8.What is the **main** source of funding for paediatric hepatitis treatment at your practice/facility?

Select one:
- ☐ Patients (i.e., self-funded)
- ☐ Government (public sector)
- ☐ Private insurance
- ☐ NGO
- ☐ Other (please specify below)

**Section C Preferences for future HCV treatment and DAA regimens**

9. How important are the following outcomes/concerns when you are considering whether to treat children and adolescents?

| | Not at all important | Low importance | Neutral | Very important | Extremely important |
|---|---|---|---|---|---|
| SVR12 (cure) | | | | | |
| Measure of liver disease (e.g. biopsy, non-invasive liver fibrosis assessments) | | | | | |
| Presence and severity of symptoms | | | | | |
| Measures of psychological well being | | | | | |
| Measures of physical function | | | | | |
| Educational attainment | | | | | |
| Long-term adverse effects | | | | | |

| | | | | | |
|---|---|---|---|---|---|
| Prevention of transmission to other persons | | | | | |
| Prevention of stigmatisation of infected children | | | | | |

10a. If DAAs were available for treatment of HCV infection in children and adolescents in your practice/facility, how likely would you be to treat children in the following age groups:

| | Extremely unlikely | Unlikely | Neutral | Likely | Extremely likely |
|---|---|---|---|---|---|
| 0 to < three years old | | | | | |
| 3 to < 6 years old | | | | | |
| 6 to < 12 years old | | | | | |
| 12 to <18 years | | | | | |

| | |
|---|---|
| 10b.Please specify reasons for your preferences for not treating a particular age group | Open-ended |

11a. If DAAs were available for treatment of HCV infection in children and adolescents in your practice/facility, which DAA regimens would you prefer to use in the following age groups:

| | 0 to < three years old | 3 to < 6 years old | 6 to < 12 years old | 12 to <18 years |
|---|---|---|---|---|
| Sofosbuvir + interferon | | | | |
| Sofosbuvir + ribavirin | | | | |
| Sofosbuvir/daclatasvir | | | | |
| Sofosbuvir/ledipasvir | | | | |
| Sofosbuvir/velpatasvir | | | | |
| Sofosbuvir/velpatasvir/voxilaprevir | | | | |
| Glecaprevir/pibrentasvir | | | | |

| | |
|---|---|
| 11b. Please specify reasons for your preferences | Open-ended |
| 12. Which children would you prioritise for treatment with DAAs? | Open-ended |
| 13. Which children would you not wish to treat with DAAs? | Open-ended |
| 14. In your opinion, what is the threshold age at which treatment of children with HCV should be recommended in treatment guidelines?<br><br>Please select one | ☐ All age groups should be treated<br>☐ three years and above<br>☐ 6 years and above<br>☐ 12 years and above<br>☐ Other, please specify |

| **Section D Barriers and solutions to promoting access to HCV treatment for children and adolescents** | |
|---|---|
| 15.What barriers do you face in your practice/facility in treating children and adolescents with HCV infection?<br><br>Select all that apply | ☐ Lack of national policies and guidelines<br>☐ DAAs are not available<br>☐ DAAs are available but not registered for use in children and adolescents<br>☐ DAAs are available but not free (patients have to pay to receive treatment)<br>☐ DAAs are available but paediatric formulations are not available<br>☐ Lack of awareness among health professionals<br>☐ Lack of awareness among patients<br>☐ Fear/misconceptions about the treatment<br>☐ Others (please specify) |
| 16.What are your suggestions to facilitate access and uptake of HCV testing and treatment for children and adolescents? | Open ended questions |
| 17. Is there anything else you would like to tell us about promoting access to HCV testing and treatment of children and adolescents in your country? | |

| | |
|---|---|
| If you are interested in collaborating in sharing of anonymized individual patient data to future studies, please enter your name and email address | Respondent name<br>Respondent email |

By clicking the ‘Submit’ button below, you are consenting to participate in this study, as it is described in the participant information sheet, which you can download here.

**We very much appreciate your time in completing this survey!**
